# Indonesia youth population norms for EQ-5D-Y-3 L, EQ-5D-Y-5 L and the PedsQL generic core scale: lower health related quality of life relates to high economic status and stress

**DOI:** 10.1186/s12889-023-16003-0

**Published:** 2023-06-12

**Authors:** Titi Sahidah Fitriana, Fredrick Dermawan Purba, Elly Stolk, Jan J. V. Busschbach

**Affiliations:** 1 Department of Psychiatry, Section Medical Psychology and Psychotherapy, Erasmus MC University Medical Center, Wytemaweg 80, Rotterdam, 3015 CN The Netherlands; 2 Faculty of Psychology, YARSI University, Jakarta, Indonesia; 3 Department of Developmental Psychology, Faculty of Psychology, Universitas Padjadjaran, Jatinangor, Indonesia; 4 The EuroQol Research Foundation, New York, USA

**Keywords:** EQ-5D-Y, PedsQL, Population norms, Health-related quality of life

## Abstract

**Background:**

The availability of population norms from generic health-related quality of life (HRQoL) instruments can support the interpretation of health outcomes. This study aimed to provide Indonesian youth population norms for the generic HRQoL measures: EQ-5D-Y-3 L, EQ-5D-Y-5 L, and the PedsQL Generic Core Scales. In addition the opportunity arising from the generation of a large representative sample was taken to explore the relationships between HRQoL, health, and socio-economic factors.

**Methods:**

A representative sample of 1103 Indonesian children (aged 8–16 years) completed EQ-5D-Y-3 L, EQ-5D-Y-5 L, the PedsQL Generic Core Scales, and questions related to demographic data and self-reported health status. A stratified quota sampling design was used to represent Indonesian children in terms of residence, age, gender, and geographical area. Family expenses per capita per month were retrieved from parents to determine a child’s economic status.

**Results:**

The total sample was representative of the Indonesian youth general population. The proportions of participants who reported problems were 43.35% (EQ-5D-Y-3 L), 44.10% (EQ-5D-Y-5 L), and 94.93% (PedsQL Generic), with 31.7% of children reporting health complaints. Older children (13–16 years) reported more problems than younger children (8–12 years). Children living in urban areas reported more problems than children living in rural areas. The lowest value health state reported was ‘12332’ (valued at 0.54), and the minimum EQ VAS score was 60.00. Moderate correlations were found between EQ-5D-Y-3 L values to EQ VAS scores and to PedsQL Total Score. Hierarchical regression analysis showed that females, older age, and having health complaints contributed to a lower level of HRQoL as measured by EQ-5D-Y-3 L values, EQ VAS, and PedsQL Total Score. Remarkably, children with high economic status had lower EQ VAS and PedsQL Total Scores. Among symptoms, ‘having stress’ had the largest influence with respect to lower EQ-5D-Y-3L values, EQ VAS, and PedsQL Total Score.

**Conclusions:**

Population norms for children’s HRQoL as measured by EQ-5D-Y-3 L, EQ-5D-Y-5 L, and the PedsQL Generic Scales are now available for Indonesia. Age, gender, economic status, and health complaints were related to children’s HRQoL. These results provide a basis for health studies and health policy for the youth population of Indonesia.

**Supplementary Information:**

The online version contains supplementary material available at 10.1186/s12889-023-16003-0.

## Background

The development and use of pediatric health-related quality of life (HRQoL) measures has increased in the past decade [1, 2], including in Indonesia. HRQoL is a multidimensional construct including factors related to individual health, and excludes non-health quality of life factors such as the environment [3, 4]. When operationalizing HRQoL into questionnaires, differentiation in instrument specificity enables instruments to be divided into generic and disease-specific. Generic HRQoL instruments, such as EQ-5D-Y or PedsQL, have a broad health domain, resulting in an ability to detect only important changes, while disease-specific measures have more sensitivity in picking up health complaints [5, 6]. Which instrument to use depends on the study aim.

The interpretability of health status instrument scores leaves room for improvement: in most countries, norm scores for pediatric instruments are lacking, which means that it is unknown what score represents ‘normal’ HRQol or health impairment. Development of norm scores allows the results obtained from an individual child or group of children to be compared with the distribution of scores in the normal population as a ‘benchmark’ of the healthy population with similar relevant characteristics, such as age and gender [7]. Such comparisons enable researchers to assess the burden of diseases in terms of HRQoL and to compare this burden relative to the normal population or to other disease groups [8, 9]. Population scores can also facilitate evaluation of treatment effectiveness and deliver effect sizes comparable over interventions and patient groups [10, 11]. The closer post-treatment HRQoL scores approach population health scores, the more the treatment makes the patients’ HRQoL resemble the HRQoL of the normal population. Hence the availability of population scores, better known as ‘population norms’, can inform clinical decision-making, public healthcare programs, and health policy.

Indonesia currently has norm scores available for two HRQoL measures: EQ-5D-5 L [12] and WHOQOL-BREF [12]. However, these were developed for use in adults, while Indonesia has a relatively young population with a median age of 29.7, and 32.4% of the population younger than 19 [13, 14]. Thus it is pertinent to generate population norms for important measures of child health. Currently, there are two generic HRQoL instruments that have been tested psychometrically in pediatric patients in Indonesia: the PedsQL Generic Core Scales [15] and the EQ-5D-Y-3 L and its extension EQ-5D-Y-5 L [16]. Norm scores are not yet available for these instruments.

The objective of this study was to derive population norms for EQ-5D-Y-3 L, EQ-5D-Y-5 L, and PedsQL Generic Core Scales for Indonesian children aged 8–16 years, differentiating these norms by demographic characteristics of participants such as age and gender. We also aimed to explore the relationships between the HRQoL scores for the general population and participants’ characteristics such as age, gender, residence, economic status, region, and health complaints.

## Methods

### Sampling

We used a stratified quota sampling design to create a sample of children representing the Indonesian population of children in terms of residence, age, gender, and geographical area. Quotas were defined based on the most recent Indonesian Bureau of Statistic census [17]. Stratification was set geographically based on population size on each island: 57% collected from Java, 22% from Sumatra, 7% from Kalimantan, 6% from Sulawesi, and 8% from other parts of Indonesia. Quota sampling was calculated based on residence (urban/rural), age (8–12/13–16), and gender (male/female), regardless of the island. Quota completion was done continuously to ensure the required sample was fulfilled. When the quota for a specific group was full, no further participant recruitment with the corresponding criteria was done on the subsequent data collection time.

To keep the study feasible, children were recruited through the school since the targeted children were in a school age (8 to 16 years). Schools were participated voluntary, and within these schools, classes were selected based on availability. Thus, the fulfillment of the sampling criteria was done on the basis of the characteristics of the children, and not on the characteristics of the schools. Data was collected in 26 different schools located in different areas in Indonesia from July to December 2019. Children with matched criteria were provided by the school on the collection day. Because the school participation rate in Indonesia is 99% [17], complementary door-to-door interviews were conducted to represent the 1% of Indonesians not attending school. Prior to participation, informed consent was sent to the parents. Only participants with signed informed consent from their parents on the day of collection participated in the survey. All procedures performed in this study were in accordance with the ethical standards of the Health Research Ethics Committee, YARSI University, Jakarta, Indonesia (ethics approval number: 115/KEP-UY/BIA/VII/2019).

### Data collection

All children participating in the survey were administered the instruments described below and asked a set of demographic questions, such as date of birth, gender, place of residence, and education. Children were also asked whether they had health complaints on the collection date. Per capita expenditure was used to assess economic status following the standard practice of the National Bureau of Statistics [17]. Expenses per capita per month were retrieved from the parents, along with informed consent.

#### EQ-5D-Y-3 L™

The EQ-5D-Y instrument comprises a short questionnaire entitled the descriptive system, and a visual analogue scale, the EQ VAS. This descriptive system includes 5 dimensions: mobility (walking about), looking after myself, doing usual activities, having pain or discomfort, and feeling worried, sad, or unhappy. The response format has 3 severity levels: no problems, some problems, and a lot of problems [18]. The responses on the descriptive system can be expressed by a 5-digit code that uniquely represents each health state. For example, the EQ-5D-Y-3 L health state 12,331 describes someone with no problems with mobility, some problems with looking after myself, a lot of problems with doing usual activities and pain/discomfort, and no problems of feeling worried, sad, or unhappy. Each health state has a value attached based on the available value set from the respective country. These values reflect the preferences of the general population for health states and are measured on a scale that is anchored at 1 (full-health) and 0 (dead). Negative value is possible and represents a health state considered worse than dead. For Indonesian EQ-5D-Y-3 L value sets, please refer to Fitriana et al. [19].

#### EQ-VAS

The EQ VAS is a part of the EQ-5D instrument. It invites a person to rate their current overall state of health on a standard, vertical, visual analogue scale. The EQ VAS ranges from 100 (the best health state you can imagine) to 0 (the worst health state you can imagine). EQ VAS scores are collected in addition to responses on the EQ-5D descriptive system in order to produce a rating (score) for their own health.

#### PedsQL™ 4.0 generic core scales

The PedsQL™ 4.0 Generic Core Scales (Copyright © 1998 JW Varni, Ph.D.) is a self-report questionnaire that consists of 23 items divided into 4 dimensions: physical, emotional, social, and school [20, 21]. For interpretation, there are 3 summary scores, physical health (score on the physical dimension), psychosocial health (score on the latter 3 dimensions), and Total Score. The questionnaire consists of 5 level responses from 0 to 4, where 0 means ‘never a problem’ and 4 means ‘almost always a problem’. Scores are reversed and linearly transformed to a 0-100 scale (0 = 100, 1 = 75, 2 = 50, 3 = 25, 4 = 0). Average scores per dimension are computed, where higher scores indicate better HRQoL.

#### EQ-5D-Y-5 L

EQ-5D-Y-5 L has the same dimensions and wordings as EQ-5D-Y-3 L with an extension of response format to 5 severity levels; no problems, a little bit of a problem, some problems, a lot of problems, and cannot/extreme problems [22]. In the EQ-5D-Y-5 L version, the added levels are level 3 (some problems) and level 4 (a lot of problems). EQ-5D-Y-5 L is still an experimental version for which further testing is needed to assess the benefit of level structure expansion from 3 to 5. However, evidence suggests that EQ-5D-Y-5 L has better measurement properties than EQ-5D-Y-3 L [16, 23, 24]. At present we are unable to report the same range of analysis as EQ-5D-Y-3 L due to the current status of the instrument and the unavailability of an EQ-5D-Y-5 L value set.

### Statistical analysis

#### Population norms data

The following data were compiled and analyzed. (i) Participant characteristics percentages were calculated and compared to the 2015 Indonesian census to assess the representativeness of the sample. (ii) The health states obtained from the EQ-5D-Y-3 L descriptive system were converted to health values based on the Indonesian EQ-5D-Y-3 L value set [19]. (iii) A descriptive summary of distribution scores was performed for EQ-5D-Y-3 L values, EQ VAS scores, and PedsQL Total Scores. (iv) The reported problems per EQ-5D-Y level across dimensions, EQ VAS mean scores, EQ-5D-Y-3 L mean values, and PedsQL scores were calculated based on age and gender, as these two factors are related to HRQoL in many populations [25]. (v) Further detail of participants’ characteristics such as residence, gender, age, region, and economic status were used to stratify the mean and standard deviation (SD) for the HRQoL scores as measured by EQ VAS, EQ-5D-Y-3 L values, and PedsQL Generic Core Scales. (vi) Differences in HRQol across demographic subgroups (i.e., rural vs. urban, female vs. male) were tested using the Welch’s unequal variances t-test, given the skewed data and differences variances. ANOVA was used to compare mean scores for more than two groups, such as region and economic status. (vi) The top 5 and the worst EQ-5D-Y-3 L health states were calculated, together with correlations between the EQ-5D-Y-3 L values, EQ VAS, and PedsQL total score.

#### The relation between background variable and HRQoL

Hierarchical regression analysis was conducted to explore the influence of participants’ demographic characteristics and health factors on EQ-5D-Y-3 L health values, EQ VAS and PedsQL total score. Hierarchical regression is an appropriate analytical approach when variance on a variable being explained by predictor variables is correlated [26]. Hierarchical regression analysis considers all related variables and adds to the model at each regression step. The hierarchical regression model aims to determine whether the newly added variables show a significant improvement in the explained variance. Statistical analysis was performed using IBM SPSS Statistic version 21.0.

There were 6 variables assessed in the hierarchical model; age, gender, residence (urban/rural), economic status (low/middle/high), region (Jawa, Sumatera, Kalimantan, Sulawesi, and others), and health complaints. Age and gender are known to be variables related to health [25], hence these 2 variables acted as a basic model that takes the form of Eq. 1. Studies have found a positive association between children’s health and parental economic status [27], hence economic status was added, in addition to residence and region. Economic status was divided into low, middle, and high. Low economic status was assigned to a families’ expenses below Rp 929.000/capita/month (equal to 60 Euro, 2023). Middle-class expenses ranged from Rp 930.000 to Rp 4.497.000, while high economic-class expenses was above Rp 4.500.000,- /capita/month (290 Euro, 2023) [17]. Variables were added successively to the model in order to investigate the contribution of each factor into the model. The reported health complaints were coded and combined into five categories asthma, stress, upper respiratory problems, gastritis, and others. These five categories were used in the quantitative analysis (Eq. 2).

1 $$ {Y_i} = {\beta _0} + {\beta _1}male + {\beta _2}(age13 - 16)$$

2 $$ \begin{array}{l}{Y_i} = {\beta _0} + {\beta _1}male + {\beta _2}\left( {age13 - 16} \right) + {\beta _3}rural\\+ {\beta _4}middle + {\beta _5}high + {\beta _6}sumatera\\+ {\beta _7}kalimantan + {\beta _8}sulawesi + {\beta _9}others\\+ {\beta _{10}}Upperrespiratoryproblem + {\beta _{11}}stress\\+ {\beta _{12}}gastritis + {\beta _{13}}asthma + {\beta _{14}}others\end{array}$$

$$ {Y}_{i}$$ are the HRQoL summary scores as measured by EQ-5D-Y-3 L values, EQ VAS, or PedsQL total scores. $$ {\beta }_{0}$$ is the regression intercept, $$ {\beta }_{1}$$ to $$ {\beta }_{14}$$ are the coefficients related to the predictor variables. All variables are dummy coded where the first category acts as a reference to the other category (for example: the coefficient for male is relative to female, as female is the first category and acts as a reference).

## Results

### Participants characteristics

**Fig. 1:**
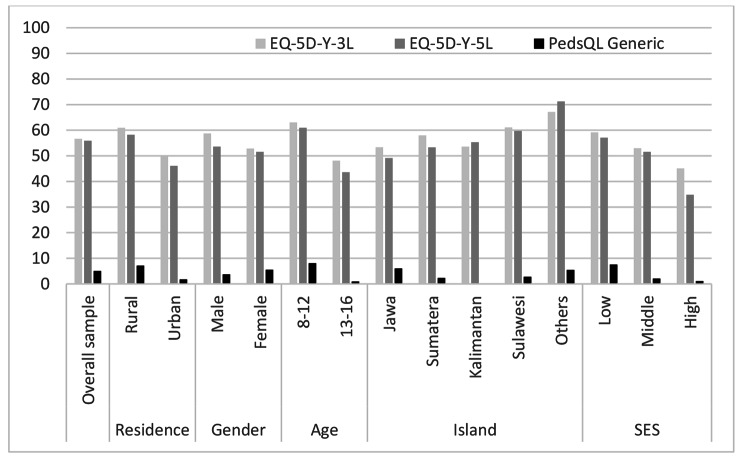
Percentage of participants reporting full-health (11,111 for EQ-5D-Y or 100 for PedsQL)

Table 1 shows the characteristics of 1103 participants collected in this study. Proportion differences between the study sample and the targeted population were less than 4%. The majority of respondents lived in Java (58.5%), had low economic status (52.9%), were healthy (68.3%), and enrolled in school (99%). Most respondents reported good health as measured by EQ-5D-Y-3 L values, EQ VAS, and PedsQL Total Scores, with the largest variability found in PedsQL and smallest in EQ-5D-Y-3 L values (S-Fig. 1, S-Fig. 2 and S-Fig. 3). The sample’s mean, median and standard deviation for EQ-5D-Y-3 L values, EQ VAS, and PedsQL Total Scores are summarized in S-Table 1. More detail concerning self-reported health is provided in supplementary file 2.

**Table 1:** Demographic characteristics of participants

Characteristics	Study Sample N = 1103 (%)		Indonesian Population (%)	Differences (%)
Residence				
Rural	589	53.4	50.2	+ 3.2
Urban	514	46.6	49.8	-3.5
Gender				
Female	543	49.2	49.7	-0.5
Male	560	50.8	50.3	+ 0.5
Age				
8–12	571	51.8	50.0	+ 1.8
13–16	532	48.2	50.0	-1.8
Region				
Java	645	58.5	56.8	+ 1.7
Sumatera	257	23.3	21.6	+ 1.7
Kalimantan	56	5.1	7.3	-2.2
Sulawesi	72	6.5	6.0	+ 0.5
Others	73	6.6	8.2	-1.6
Economic status				
Low	529	52.9	43.3	NA
Middle	287	28.7	56.5	NA
High	184	18.4	0.2	NA
*Missing*	103	9.3	-	NA
Education				
Elementary Middle High Quitting	533 250 309 11	48.3 22.7 28.0 1.0	62.0 23.4 14.6 1.0	NA NA NA NA
Health				
With symptom	350	31.7	-	NA
Healthy	753	68.3	-	NA

NA = not Applicable

### Population norms for EQ-5D-Y-3 L and EQ-5D-Y-5 L

The proportions of reported problems per level in EQ-5D-Y-3 L and EQ-5D-Y-5 L stratified by age, gender, and other demographic characteristics are shown in Supplementary file 2 (S-Table 2 to S-Table 4). As expected in a general population sample, a substantial number of respondents reported no problems on any of the 5 dimensions, ranging from 71.1 to 98.4% for EQ-5D-Y-3 L and 69.5–97.8% for EQ-5D-Y-5 L (S-Table 2). Across age and gender, the fewest problems were reported in ‘looking after myself’. Older females reported problems more frequently in the ‘feeling worried/sad/unhappy’ dimension than the other groups, both in EQ-5D-Y-3 L and EQ-5D-Y-5 L (S-Table 2 and S-Table 3). For all demographic groups, the proportion of problems with ‘pain/discomfort’ and ‘feeling worried, sad, or unhappy’ was higher than problems on the other dimensions (S-Table 4).

Table 2 shows the mean EQ VAS and EQ-5D-Y-3 L values for the total population and for the different demographic characteristics. The mean EQ VAS for the total population was 89.35 (SD 11.81). The EQ VAS mean scores were significantly lower in urban compared to rural, lower in females than males, and lower in those 13–16 years old than 8–12 years. Based on region, children living in Java and Sumatera reported significantly lower EQ VAS scores than those living in Sulawesi and others. In terms of economic status, children with high economic status showed lower EQ VAS scores than children from low and middle economic backgrounds. For EQ-5D-Y-3 L values, differences appeared significant between gender (lower values for females) and age (lower for 13–16) (Table 2), but not on the other demographic factors. The mean values for EQ VAS and EQ-5D-Y-3 L by age and gender are summarized in Table 3.

**Table 2:** Mean scores of EQ-VAS, EQ-5D-Y-3 L values, and PedsQL Generic

Demographic		N	EQ-VAS		EQ-5D-Y-3 L values		PedsQL Psychosocial		PedsQL Physical		PedsQL Total	
			Mean	SD	Mean	SD	Mean	SD	Mean	SD	Mean	SD
All participants		1103	89.35	11.81	0.96	0.07	77.60	14.22	85.47	12.74	80.33	12.76
Residence	Rural	589	**90.89**	11.41	0.97	0.05	78.99*	14.62	86.02	12.91	81.43*	13.18
	Urban	514	**87.57**	12.02	0.97	0.05	76.01*	13.58	84.83	12.52	79.08*	12.17
Gender	Female	543	**87.80**	12.78	**0.97**	0.06	76.00*	14.68	84.10*	13.37	78.82*	13.25
	Male	560	**90.84**	10.58	**0.97**	0.05	79.16*	13.58	86.79*	11.95	81.80*	12.10
Age	8–12	571	**91.21**	11.64	**0.98**	0.05	81.49*	14.04	88.18*	11.93	83.81*	12.44
	13–16	532	**87.35**	11.66	**0.97**	0.05	**73.45**	13.21	82.55*	12.94	76.62*	12.04
Region	Java	645	**88.82**	11.94	0.96	0.07	76.62	14.53	84.83	12.55	79.46	12.83
	Sumatera	257	**88.42**	11.89	0.97	0.07	77.94	13.68	84.98	13.34	80.39	12.65
	Kalimantan	56	90.04	13.36	0.95	0.09	78.04	15.19	86.38	14.11	80.94	13.95
	Sulawesi	72	**92.97**	9.37	0.97	0.06	77.20	14.13	85.94	12.87	80.24	13.00
	Others	73	**93.12**	9.93	0.98	0.05	85.11*	10.04	91.65*	9.08	87.39*	8.89
Economic status	Low	529	**90.33**	11.20	0.96	0.07	78.52*	14.94	85.98	13.10	81.10*	13.33
	Middle	287	**89.02**	12.59	0.96	0.07	76.72	13.51	85.25	12.03	79.69	12.16
	High	184	**85.15**	12.37	0.95	0.08	74.85*	12.96	83.49	12.54	77.85*	11.82

SD : standard deviation

**Table 3:** Self-reported EQ-VAS, EQ-5D-Y-3 L values, and PedsQL Generic by age group and gender

Variable	Total Sample		Male				Female			
			8–12 years		13–16 years		8–12 years		13–16 years	
	Mean	SD	Mean	SD	Mean	SD	Mean	SD	Mean	SD
EQ-VAS	89.35	11.81	92.91	10.57	88.57	10.13	89.41	12.45	86.12	12.93
EQ-5D-Y-3 L values	0.96	0.07	0.98	0.05	0.97	0.04	0.98	0.98	0.96	0.06
PedsQL Total	80.33	12.76	84.39	11.92	78.99	11.69	83.20	83.20	74.23	11.95
PedsQL Physical	85.47	12.74	88.61	11.49	84.78	12.16	87.72	87.72	80.31	13.33
PedsQL Psychosocial	77.60	14.22	82.16	13.51	75.89	75.89	80.78	14.56	70.99	13.07

SD : standard deviation

The percentages of participants reporting full health (no problems in all dimensions: ‘11111’) in EQ-5D-Y were lower for people living in urban areas, females, older children (13–16 years), living in Java, and for those with high economic status (Fig. 1). More than half of the sample reported no problem on the 5 dimensions (56.65% for EQ-5D-Y-3 L and 55.90 for EQ-5D-Y-5 L) (Fig. 1). 33 out of 243 possible health states of EQ-5D-Y-3 L were reported by the participants. The top 5 and the worst health states reported are presented in Table 4. Respondents that rated themselves as ‘11111’ assigned a mean score of 92.64 on the EQ VAS scores (Table 4). The lowest health state reported was ‘12332’ with a value of 0.54, an EQ VAS score of 60.00, and a PedsQL Total Scores of 48.00. There was a moderate and positive correlation between EQ-5D-Y-3 L values and EQ VAS scores (rho = 0.38, p < 0.01).

**Table 4:** Correlation between EQ-5D-Y-3 L health states with EQ-VAS and PedsQL

Health state	N	%	Cum. prob	EQ-5D-Y-3 L values	EQ-VAS				PedsQL Total Scores			
					Min	Max	Mean	SD	Min	Max	Mean	SD
11,111	616	55.85	100.00	1	35	100	92.64	9.81	51.00	100.00	85.51	11.50
11,121	115	10.43	44.15	0.98	40	100	87.17	11.53	53.00	100.00	77.68	12.49
11,112	107	9.70	33.72	0.98	45	100	87.83	10.75	36.00	96.00	76.07	10.26
11,122	105	9.52	24.02	0.93	30	100	82.63	13.22	42.00	95.00	70.75	10.77
11,222	47	4.26	14.50	0.83	40	100	80.23	13.83	37.00	90.00	64.08	10.16
12,332	1	0.09	0.09	0.54	-	-	60.00	-	-	-	48.00	-
Spearman’s rho							.38**				0.44**	

SD : standard deviation

**correlation is significant at the 0.01 level.

The results of the hierarchical regression analysis to evaluate the prediction of EQ-5D-Y-3 L values and EQ VAS from gender, age, residence, economic status, and region can be seen in Table 5. In this analysis, age and gender contributed significantly to the values of EQ-5D-Y-3 L. The other demographic variables in models 2,3 and 4 did not contribute significantly to the prediction of EQ-5D-Y-3 L values. The EQ VAS prediction model showed that, apart from residence, the demographic characteristics of participants - gender, age, economic status, and region - contributed significantly to the EQ VAS score. Higher socio-economic status was associated with lower EQ VAS scores, even after adjustment of other related factors.

**Table 5:**
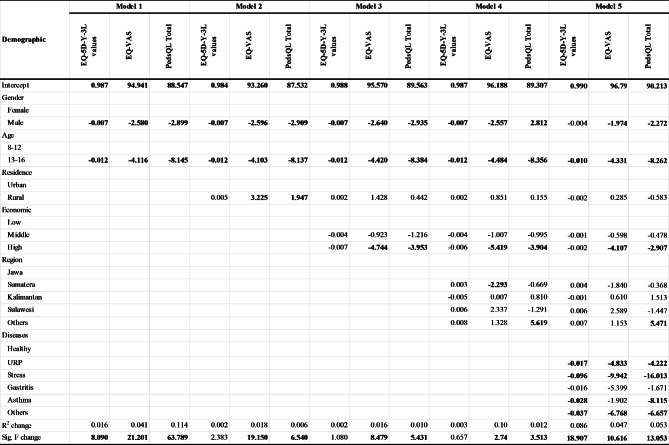
Hierarchical regression model for EQ-5D-Y-3 L values, EQ-VAS, and PedsQL Generic Core Scale

Table 5 shows the contribution of health complaints to the HRQoL scores of the participants. After controlling for age and gender, having health complaints was significantly related to EQ-5D-Y-3 L values and the EQ VAS scores of participants. Among complaints, ‘stress’ had the largest influence on the reduction of EQ-5D-Y-3L values and EQ VAS scores.

### Population norms for PedsQL generic core scale

As expected in a general population sample, most of the respondents reported high scores on the PedsQL total score (mean = 80.33, SD = 12.76) (S-Fig. 3). The proportions of reported problems per summary score and Total Score are reported in Supplementary file 2 (S-Table 4). In the total sample, the percentage of children who reported psychosocial problems was higher than for physical problems (94.1% vs. 80.1%). Children living in urban areas tended to report more problems in the psychosocial and physical dimensions compared to children living in rural areas. Similarly, older age [13–16] children reported more problems than younger children [8–12] (S-Table 4).

Table 2 shows the PedsQL means for the total sample and for the summary scales of the different sociodemographic characteristics. In the total sample, the psychosocial scale mean was 77.60 (SD 14.22), the physical scale mean was 85.47 (SD 12.74), and the Total Score mean was 80.33 (SD 12.76). The summary and the PedsQL Total Score were significantly lower in urban residence than in rural, in female compared to male, and in older children (13–16 years) compared to younger [8–12]. Participants living in ‘other areas’ had significantly higher PedsQL scores compared to the participants living in Java, Sumatera, Kalimantan and Sulawesi. The PedsQL Generic Core Scale means by age and gender are summarized in Table 3, which shows that male and young participants had the highest scores. The percentages of participants reporting full health (score of 100) on the PedsQL Generic Core Scales ranged from 0.00 to 8.06% (Fig. 1). The PedsQL Total Scores were lower for: children living in urban areas, males, older children(age 13–16 years), living in Kalimantan, and with high economic status. The means of PedsQL Total Score, PedsQL Physical and PedsQL Psychosocial by age and gender are summarized in Table 3. Table 4 shows a moderate and positive correlation between EQ-5D-Y-3 L values and to PedsQL Total Score (rho = 0.44, p < 0.01). A hierarchical regression model was conducted to predict the PedsQL Total Score from gender, age, residence, economic status and region (Table 5). Apart from residence (urban/rural), it can be inferred that participants’ demographic backgrounds increased the model’s prediction for the PedsQL Total Score. Higher socio-economic status was associated with lower HRQol scores, even after adjustment for other related factors. Table 5 shows the contribution of health complaints to participant HRQoL scores. After controlling for age and gender, the factor ‘health complaints’ related negatively to the PedsQL Total Score. Among complaints, ‘stress’ had the largest influence on the reduction of the PedsQL Total Score.

## Discussion

In this article we present Indonesian population norms for 2 versions of EQ-5D-Y, and for the PedsQL Generic Core Scales. The EQ-5D-Y norms were complemented with EQ VAS scores, and values for EQ-5D-Y-3 L reported by children. Data were obtained from 5 islands in Indonesia utilizing quota sampling based on gender, age, and residence, in order to represent Indonesian children. The differences of less than 4% between the sample results and those for Indonesia general population indicate that these norms are representative for this population, and thus valid for use in clinical research and health outcomes evaluation.

Despite the study being conducted among the general youth population, the proportions of participants reporting HRQoL problems ranged from 43.35% (EQ-5D-Y-3 L) to 94.93% (PedsQL Total Score). The proportion of reported problems on the PedsQL instrument can be considered as very high if it is taken into account that only 31.7% of participants reported health complaints. These findings imply that PedsQL can be considered as more sensitive to problems whether the problems are exclusively related to illness or not.

The finding that children living in urban areas reported more problems in the pain/discomfort and worried/sad/unhappy dimensions than children living in rural areas differed from earlier studies. It has been well-documented that urban residents have better access to healthcare facilities that later lead to lower morbidity and mortality [28–30]. However, rurality per se does not necessarily lead to urban-rural disparities. There are embedded characteristics of rural areas that are related to these disparities, such as socio-economic factors, poorer service availability, hazardous environments, and so forth [31]. When these major risk determinants are well-managed, disparities might not arise, especially in children without any illness. On the other hand, living in rural areas might bring health advantages for children in terms of green spaces available for leisure and physical activities which can affect children’s health and well-being [32–34]. Our results suggest that health policy and city design should also take into account that currently cities in Indonesia may not provide optimal environments in which the next generation can grow up.

Our study showed that females reported notable differences in the worried/sad/unhappy dimension compared to males. This finding is in line with previous study in adult where Indonesian females reported more health problems than males [12]. Differences between the health status of males and females is common in adult studies [35, 36], although this is not always the case in children. Recent studies have reported no differences between male and female health status in children [37, 38], whilst other studies have shown differences between the genders [39, 40]. Likewise, a large gap was observed between children aged 8–12 and 13–16 in terms of reported pain/discomfort, and worried/sad/unhappy on EQ-5D-Y, and psychosocial problems, physical score, and Total Scores as measured by PedsQL. Adolescence is known to be a challenging phase where substantial physical, psychological and behavioral changes are experienced [41]. Due to these changes, mental health problems may emerge in late childhood and early adolescence. Our results stress that these mental problems do need attention and treatment, even in a developing country such as Indonesia [42].

Regardless of which instrument was used, the hierarchical regression model showed that gender and age contributed to the participants’ HRQoL as measured by EQ-5D-Y-3 L values, EQ VAS, and PedsQL Generic Scores. Region and economic status correlated negatively to EQ VAS, and PedsQL Generic Scores, but not with EQ-5D-Y-3 L values. Given that EQ-5D-Y-3 L correlated well with own reported health, this suggests that EQ VAS and PedsQL may measure more factors beyond those most people would consider ‘health problems’. In model 2, all three predictors, gender, age, and residence, explained a part of the variation in EQ VAS and PedsQL Total Scores. However, when economic status was added to the model, residence was no longer a significant predictor of the dependent variables. This result implied that economic status was the underlying cause of health disparities between urban and rural areas.

Contrary to a common belief that higher economic status correlates positively with health related quality of life, our findings showed that being of high economic status reduced the overall perceived health score as measured by EQ VAS and PedsQL Generic. It might be wise to explain these findings from the perspectives of vulnerability paradox. Previous research has identified that lower vulnerability at the country level is accompanied by a higher prevalence in a variety of mental health problems in national population [43]. More affluent countries are characterized by higher levels of individualism and a lower levels of protective social support that leads to a higher level of health problems [44]. This rationale is inline with our findings: Children with high economic status usually enroll in better schools with greater academic demands. This academic demands leads to a higher competition that might ended by higher individualism and lower social support. The latter factor is one of the leading cause of stress in young people that has an impact on the mental health and well-being of children [45, 46]. This reasoning is supported by our findings, where self-reported stress had the highest impact on children’s HRQoL compared to other health complaints. One should note that the generalizability of our finding might be limited since economic status and school selection was not part of the study’s quota sampling. Thus, the economic status and participated school were not representative of the general population which may lead to a biased selection. 97% (97.8%) of participants with high economic status lived in Java and urban areas.

Our study analyzed the differences in HRQoL scores between children who reported health complaints and children who did not. The findings showed that reported health complaints contributed negatively to childrens’ HRQoL as measured by EQ-5D-Y-3 L values, EQ VAS, and PedsQL Generic Scores. Stress had the largest impact in reducing the HRQoL scores as measured by these 3 same indicators. This finding reflects the importance of mental health to childrens’ health and well-being. It might be of interest to incorporate the other potential covariates of HRQoL like physical activity and parents’ status in future studies, to provide a more detail study of the possible influences on quality of life.

## Conclusion

In this article we present population norms for Indonesia for three generic HRQoL questionnaires, the EQ-5D-Y (both versions) and PedsQL Generic Core Scale for the first-time. Age, female, and economic status were related negatively to children’s HRQoL. Contrary to common belief, children with high economic status had lower HRQoL scores compared to others. Health complaints played a role in reducing HRQoL scores among children, with stress as the most significant influencing factor. Despite the relatively modest sample size, we believe our study results to be sufficiently representative in estimating population norms validly according to sex, age, and residence for Indonesia at a national level. These results provide a sufficient basis for health research and decision-making with respect to young participants.

## Electronic supplementary material

Below is the link to the electronic supplementary material.

Supplementary Material 1

Supplementary Material 2

## Data Availability

Data of the present study is belong to the authors. Any request to access the data can be sent to the corresponding author.

## List of abbreviations

HRQoL: Health-Related Quality of Life
