# Supplementary material for: Indonesia youth population norms for EQ-5D-Y-3 L, EQ-5D-Y-5 L and the PedsQL generic core scale: lower health related quality of life relates to high economic status and stress
Source: BMC Public Health. 2023 Jun 12;23:1124. doi: 10.1186/s12889-023-16003-0 (PMC10262504; doi:10.1186/s12889-023-16003-0)
Supplement: Supplementary file 1 — Supplementary Material 1 [file 12889_2023_16003_MOESM1_ESM.docx]

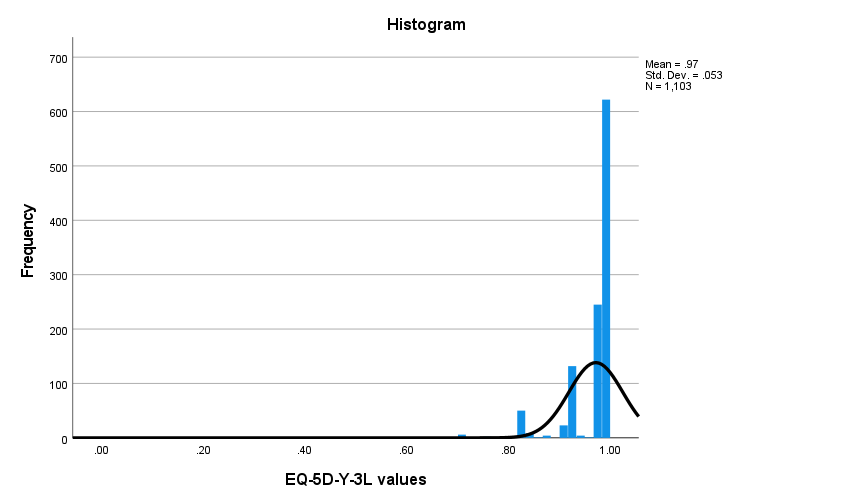


**S-Figure 1** Distribution of EQ-5D-Y-3L values (n=1.103)


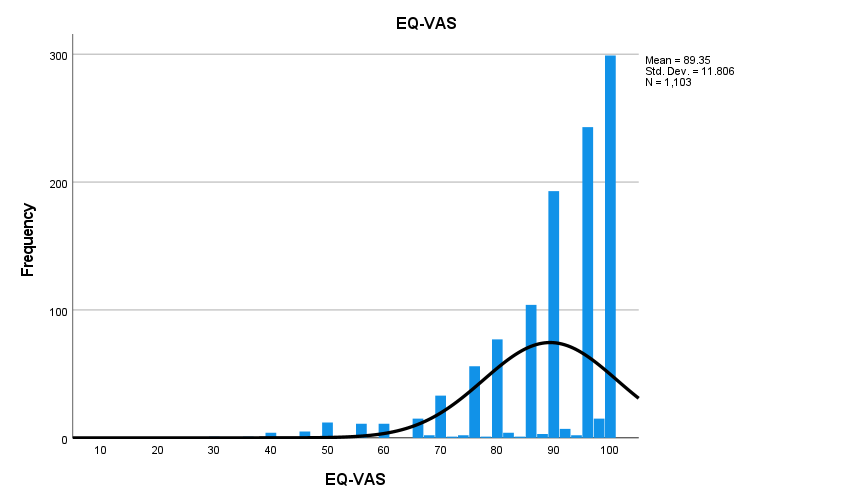


**S-Figure 2** Distribution of EQ VAS scores (n=1.103)


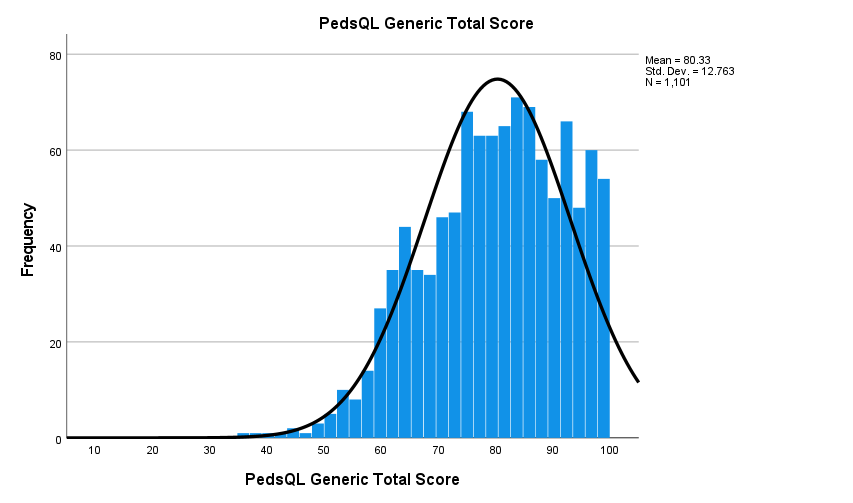


**S-Figure 3** Distribution of PedsQL Generic Total Scores (n=1.101)

**S-Table 1** Summary of mean, median, and SD of EQ-5D-Y-3L values, EQ VAS, and PedsQL Total Scores

| Variables | Mean | Median | SD |
| --- | --- | --- | --- |
| Health values EQ-5D-Y-3L | .97 | 1.00 | .05 |
| EQ-VAS | 89.35 | 95.00 | 11.81 |
| PedsQL Total Scores | 80.33 | 81.52 | 12.76 |

*SD* standard deviation
