# Supplementary material for: Indonesia youth population norms for EQ-5D-Y-3 L, EQ-5D-Y-5 L and the PedsQL generic core scale: lower health related quality of life relates to high economic status and stress
Source: BMC Public Health. 2023 Jun 12;23:1124. doi: 10.1186/s12889-023-16003-0 (PMC10262504; doi:10.1186/s12889-023-16003-0)
Supplement: Supplementary file 2 — Supplementary Material 2 [file 12889_2023_16003_MOESM2_ESM.docx]

| **S-Table 2** Frequencies of item responses in EQ-5D-Y-3L | | | | | |
| --- | --- | --- | --- | --- | --- |
| **Dimension** | **Total Sample** | **Male** | | **Female** | |
|  |  | **8-12 years** | **13-16 years** | **8-12 years** | **13-16 years** |
| Mobility |  |  |  |  |  |
| No problems  Some problems  A lot of problems | 1073 (97.3)  29 (2.6)  1 (0.1) | 284 (96.9)  8 (2.7)  1 (0.3) | 262 (98.1)  5 (1.9)  - | 270 (97.1)  8 (2.9)  - | 257 (97.0)  8 (3.0)  - |
| Looking after myself |  |  |  |  |  |
| No problems  Some problems  A lot of problems | 1085 (98.4)  18 (1.6)  - | 286 (97.6)  7 (2.4)  - | 263 (98.5)  4 (1.5)  - | 272 (97.8)  6 (2.2)  - | 264 (99.6)  1 (0.4)  - |
| Doing usual activities |  |  |  |  |  |
| No problems  Some problems  A lot of problems | 973 (88.2)  128 (11.6)  2 (0.2) | 257 (87.7)  36 (12.3)  - | 234 (87.6)  33 (12.4)  - | 248 (89.2)  29 (10.4)  1 (0.4) | 234 (88.3)  30 (11.3)  1 (0.4) |
| Pain/discomfort |  |  |  |  |  |
| No pain  Some pain  A lot of pain | 784 (71.1)  316 (28.6)  3 (0.3) | 223 (76.1)  70 (23.9)  - | 185 (69.3)  82 (30.7)  - | 217 (78.1)  60 (21.6)  1(0.4) | 159 (60.0)  104 (39.2)  2 (0.8) |
| Worried/sad/unhappy |  |  |  |  |  |
| Not worried  A bit worried  Very worried | 799 (72.4)  297 (26.9)  7 (0.6) | 241 (82.3)  50 (17.1)  2 (0.7) | 193 (72.3)  72 (27.0)  2 (0.7) | 217 (78.1)  60 (21.6)  1 (0.4) | 148 (55.8)  115 (43.4)  2 (0.8) |

| **S-Table 3** Frequencies of item responses in EQ-5D-Y-5L | | | | | |
| --- | --- | --- | --- | --- | --- |
| **Dimension** | **Total Sample** | **Male** | | **Female** | |
|  |  | **8-12 years** | **13-16 years** | **8-12 years** | **13-16 years** |
| Mobility |  |  |  |  |  |
| No problems  A little bit of a problem  Some problems  A lot of problems  Cannot | 1063 (96.4)  40 (3.6)  -  -  - | 281 (95.9)  12 (4.1)  -  -  - | 256 (95.9)  11 (4.1)  -  -  - | 268 (96.4)  10 (3.6)  -  -  - | 258 (97.4)  7 (2.6)  -  -  - |
| Looking after myself |  |  |  |  |  |
| No problems  A little bit of a problem  Some problems  A lot of problems  Cannot | 1079 (97.8)  19 (1.7)  2 (0.2)  2 (0.2)  1 (0.1) | 284 (96.9)  6 (2.0)  -  2 (0.7)  1 (0.3) | 264 (98.9)  3 (1.1)  -  -  - | 269 (96.8)  9 (3.2)  -  -  - | 262 (98.9)  1 (0.4)  2 (0.8)  -  - |
| Doing usual activities |  |  |  |  |  |
| No problems  A little bit of a problem  Some problems  A lot of problems  Cannot | 967 (87.7)  126 (11.4)  8 (0.7)  -  2 (0.2) | 261 (89.1)  30 (10.2)  -  -  2 (0.7) | 225 (84.3)  41 (15.4)  1 (0.4)  -  - | 247 (88.8)  29 (10.4)  3 (2.0)  -  - | 234 (88.3)  26 (9.8)  5 (1.9)  -  - |
| Pain/discomfort |  |  |  |  |  |
| No pain  A little bit of pain  Some pain  A lot of pain  Extreme pain | 767 (69.5)  313 (28.4)  23 (2.1)  -  - | 220 (75.1)  68 (23.2)  5 (1.7)  -  - | 176 (65.9)  87 (32.6)  4 (1.5)  -  - | 218 (78.4)  57 (20.5)  3(1.1)  -  - | 153 (57.7)  101 (38.1)  11 (4.2)  -  - |
| Worried/sad/unhappy |  |  |  |  |  |
| Not worried  A little bit worried  Quite worried  Really worried  Extremely worried | 788 (71.4)  269 (24.4)  35 (3.2)  10 (0.9)  1 (0.1) | 237 (80.9)  45 (15.4)  8 (2.7)  2 (0.7)  1 (0.3) | 178 (66.7)  77 (28.8)  9 (3.4)  3 (1.1)  - | 228 (82.0)  48 (17.3)  1 (0.4)  1 (0.4)  - | 145 (54.7)  99 (37.4)  17 (6.4)  4 (1.5)  - |

| **S-Table 4** Percentage of participants reporting any problem in each dimension | | | | | | | | | | | | | | | | | |
| --- | --- | --- | --- | --- | --- | --- | --- | --- | --- | --- | --- | --- | --- | --- | --- | --- | --- |
| **Demographic** | | **N** | **Mobility** | | **Looking after myself** | | **Usual Activities** | | **Pain/Discomfort** | | **WSU** | | **Psycho social** | | **Physical** | | **Total** |
|  |  |  | **EQ-5D-Y-3L** | **EQ-5D-Y-5L** | **EQ-5D-Y-3L** | **EQ-5D-Y-5L** | **EQ-5D-Y-3L** | **EQ-5D-Y-5L** | **EQ-5D-Y-3L** | **EQ-5D-Y-5L** | **EQ-5D-Y-3L** | **EQ-5D-Y-5L** | **PedsQL Generic** | **PedsQL Generic** | | **PedsQL Generic** | |
| **All participants** | | 1103 | 2.7 | 3.6 | 1.6 | 2.2 | 11.8 | 12.3 | 28.9 | 30.5 | 27.6 | 28.6 | 94.1 | 80.1 | | 95.2 | |
| Residence | Rural | 589 | 2.4 | 3.2 | 1.7 | 2.2 | 11.5 | 12.2 | 25.3 | 27.2 | 23.9 | 24.4 | 91.0 | 75.2 | | 92.7 | |
|  | Urban | 514 | 3.1 | 4.1 | 1.6 | 2.1 | 12.1 | 12.5 | 33.1 | 34.2 | 31.7 | 33.3 | 97.7 | 85.8 | | 98.1 | |
| Gender | Female | 543 | 2.9 | 3.1 | 1.3 | 2.2 | 11.2 | 11.4 | 30.8 | 31.7 | 32.8 | 31.3 | 93.6 | 80.7 | | 94.5 | |
|  | Male | 560 | 2.5 | 4.1 | 2.0 | 2.1 | 12.3 | 13.2 | 27.1 | 29.3 | 22.5 | 25.9 | 94.6 | 79.6 | | 95.9 | |
| Age | 8-12 | 571 | 3.0 | 3.9 | 2.3 | 3.2 | 11.6 | 11.0 | 22.9 | 23.3 | 19.8 | 18.6 | 89.7 | 74.4 | | 91.6 | |
|  | 13-16 | 532 | 2.4 | 3.4 | 0.9 | 1.1 | 12.0 | 13.7 | 35.3 | 38.2 | 35.9 | 39.3 | 98.9 | 86.3 | | 99.1 | |
| Region | Java | 645 | 2.5 | 3.1 | 2.0 | 3.1 | 11.6 | 13.5 | 32.7 | 33.3 | 29.6 | 31.8 | 91.9 | 81.6 | | 93.6 | |
|  | Sumatera | 257 | 3.9 | 5.4 | 0.8 | 1.2 | 10.1 | 9.7 | 25.3 | 27.6 | 27.2 | 29.2 | 97.3 | 82.9 | | 97.7 | |
|  | Kalimantan | 56 | 3.6 | 1.8 | 3.6 | 1.8 | 16.1 | 12.5 | 28.6 | 28.6 | 30.4 | 21.4 | 100.0 | 69.6 | | 100.0 | |
|  | Sulawesi | 72 | 2.8 | 4.2 | 0.0 | 0.0 | 13.9 | 13.9 | 22.2 | 22.2 | 19.4 | 20.8 | 97.2 | 75.0 | | 97.2 | |
|  | Others | 73 | 0.0 | 2.7 | 1.4 | 0.0 | 13.7 | 9.6 | 15.1 | 15.1 | 16.4 | 11.0 | 94.5 | 71.2 | | 94.5 | |
| SES | Low | 529 | 2.6 | 3.8 | 1.5 | 2.3 | 11.3 | 12.5 | 27.2 | 27.2 | 24.8 | 24.4 | 90.4 | 76.9 | | 92.2 | |
|  | Middle | 287 | 2.1 | 2.1 | 1.0 | 1.4 | 12.5 | 11.8 | 31.7 | 31.7 | 31.7 | 31.4 | 97.6 | 82.2 | | 97.9 | |
|  | High | 184 | 3.8 | 6.0 | 2.2 | 3.3 | 13.0 | 15.2 | 33.2 | 33.2 | 35.3 | 42.4 | 98.4 | 90.2 | | 98.4 | |
